# Image Sensing for Motorcycle Active Safety Warning System: Using YOLO and Heuristic Weighting Mechanism

**DOI:** 10.3390/s25237214

**Published:** 2025-11-26

**Authors:** Yaw-Jen Chang, Ming-Cheng Hsu, Wen-Yung Liang

**Affiliations:** Department of Mechanical Engineering, Chung Yuan Christian University, Chung Li District, Taoyuan City 320314, Taiwan

**Keywords:** YOLO, heuristic weighting mechanism (HWM), motorcycle, image recognition

## Abstract

This paper presents an active safety warning system for two-wheeled motorcycles that integrates YOLO v4 image recognition technology with a heuristic weighting mechanism (HWM) model to calculate risk scores and thus alert riders. The system’s analytical core is based on the NVIDIA Jetson TX2 module, with a camera mounted on the left-side rearview mirror of the motorcycle. YOLO is used to identify the type of approaching vehicle and measure the distance between the vehicle and the motorcycle. Moreover, the HWM model takes inputs such as vehicle type, spacing between the motorcycle and the vehicle, motorcycle speed, and distance from the intersection to generate potential risk scores. After training, the YOLO model for vehicle recognition achieved a mean Average Precision (mAP) of 92.78% at an Intersection over Union (IoU) threshold of 0.5. Additionally, the camera mounted at a 30° angle could clearly capture vehicles approaching from the left rear side of the motorcycle, achieving the highest vehicle recognition rate. Moreover, the HWM model generates a reasonable risk score to advise the rider to decelerate when the motorcycle is traveling at high speed with a vehicle approaching from behind, thereby reducing the risk of an accident and enhancing the safety of the motorcyclist.

## 1. Introduction

Two-wheeled motorcycles have become an important means of transportation in areas with limited land and dense populations due to their flexibility and maneuverability. However, traffic accidents often happen, particularly on highly congested roads, since motorcycles have to share the road with other vehicles [1,2,3]. Taking Taiwan as an example, there were 359,346 reported motorcycle-related accidents in 2023, accounting for 52.98% of all traffic accidents in the country and resulting in 1822 deaths. Due to the limitations of motorcycle structure and design, riders often lack adequate protection in the event of an accident. As a result, two-wheeled motorcycle riders face higher rates of injury and death than drivers of other types of vehicles, especially in collisions involving buses, trucks, and other large vehicles. This high level of vulnerability makes motorcyclists the most vulnerable group of all motorized vehicle users.

**Figure 1:**
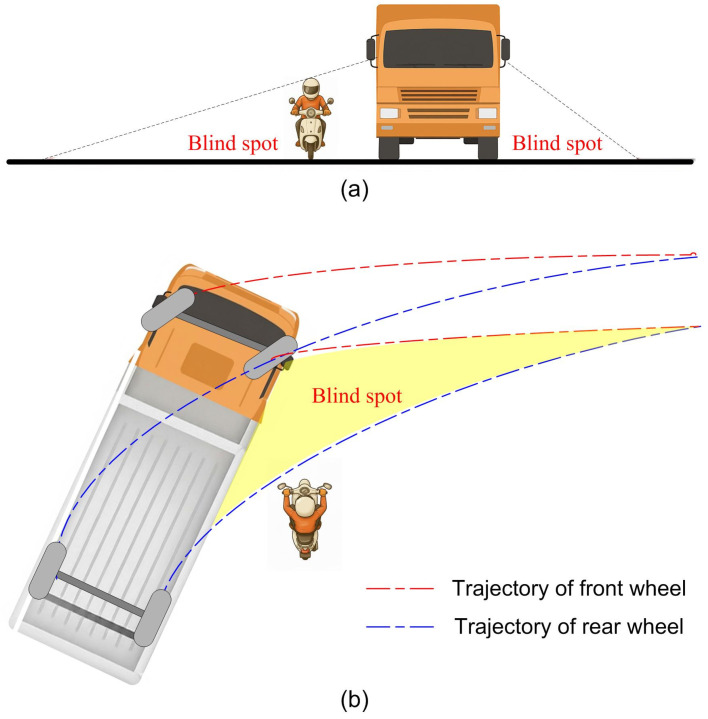
Blind spots of large vehicles: (**a**) Induced by rearview mirrors. (**b**) Caused by differences in turning radius.

Two primary causes contribute to accidents between large vehicles and two-wheeled motorcycles. The first is the problem of rearview mirrors causing blind spots for the driver. When a motorcycle travels alongside a large vehicle, the vehicle driver’s limited field of vision creates blind spots, preventing the detection of nearby motorcycles or bicycles, as illustrated in Figure 1a. If the large vehicle slightly deviates from its intended route, it may become dangerous to nearby motorcycles. The second cause is the phenomenon known as the radius difference between inner wheels that occurs when a large vehicle makes a turn. At this time, the trajectories of the front and rear wheels are different. As the wheelbase increases, the difference in turning radius between the front and rear wheels becomes greater. This difference in turning radii often results in blind spots around the vehicle, as shown in Figure 1b, obstructing the driver’s view of nearby road users. If a motorcyclist fails to recognize the vehicle’s intention to turn and inadvertently enters the blind spot, it may lead to serious or even fatal accidents. Blind spots created by differences in the inner tire radius of large vehicles are the leading cause of vehicle accident fatalities. As a result, accidents may occur when large vehicles change lanes or make turns. When a motorcycle collides with a larger vehicle, the motorcycle is often crushed under the larger vehicle, resulting in serious injury or even death.

In recent years, vehicle safety issues have attracted widespread attention and triggered a wave of research in various aspects such as cruise control [4,5], vehicular accident prevention [6,7,8], traffic flow detection [9,10], and safety assistance sensors [11]. Despite regulations requiring all large vehicles to be equipped with driving visibility assistance systems, the accident rate remains high. On the contrary, although there are relevant studies on safety auxiliary devices or systems for two-wheeled motorcycles [12,13], they are obviously insufficient, particularly in providing real-time warnings to riders.

This study employs YOLO (You Only Look Once) and the heuristic weighting mechanism (HWM) to develop an active safety warning system for motorcycles. In industry, many detection, inspection, or control technologies are based on imaging. The use of wireless signals for imaging is a state-of-the-art technology that offers sub-millisecond latency and sensing capabilities advancing toward integrated sensing and communications (ISAC) [14]. Additionally, YOLO is a real-time object detection system. The object detection process is regarded as a regression problem, which can quickly identify objects and their locations in the image. By employing a neural network applied to the entire image, the network divides the image into regions and predicts bounding boxes for each region while calculating class probabilities to predict object categories and their locations. Recently, YOLO has been widely applied to various engineering problems, such as manufacturing defect detection [15,16,17,18,19], traffic condition monitoring [20,21,22,23,24,25], robot control [26,27], image detection for medical diagnosis [28,29], etc. On the other hand, the heuristic weighting mechanism can effectively utilize domain knowledge even under limited data conditions. Heuristic rules derived from expert experience or engineering specifications are converted into feature weights, thereby adjusting the contribution of each feature to the final decision metric [30,31,32,33]. In industrial applications, HWM models have been widely adopted to encode domain knowledge as feature importance coefficients. Previous studies have shown that heuristic weighting can effectively guide the decision-making process in manufacturing quality control [34], fault diagnosis [35], and sensor fusion systems [36,37], especially in learning scenarios with limited labeled data.

In recent years, the active safety systems of motorcycles have primarily focused on antilock braking systems (ABS), autonomous emergency braking (AEB), collision avoidance, stability control, and vision assistance [38]. Among these, collision avoidance technologies commonly utilize video cameras or laser scanners to monitor the surrounding environment. Beyond merely monitoring the surrounding environment, this study utilizes YOLO to identify the types and relative distances of approaching vehicles and utilizes an HWM model to analyze the risk score. The system provides timely and early warnings to motorcyclists to help prevent potential collisions. The remainder of this paper is organized as follows: Section 2 presents the proposed methodology in detail, including vehicle type recognition using YOLO, distance estimation, and risk index evaluation using an HWM model. Section 3 discusses and analyzes the experimental results. Finally, Section 4 concludes the study and outlines potential directions for future research.

## 2. Methodology

### 2.1. Hardware

This study identifies and classifies various common four-wheeled vehicles, such as articulated trucks, buses, large and small trucks, and passenger cars, from images. The hardware utilized was the NVIDIA Jetson TX2 module, which is an embedded AI computing device that supports complex deep neural networks and can perform vehicle recognition and classification.

### 2.2. Object Detection

Object detection was conducted using the YOLO v4 open-source software. This algorithm partitions the image into an N × N grid of cells and predicts the likelihood of each grid containing a detected object. The grids with high probabilities are regarded as candidate boxes, and Non-Maximum Suppression (NMS) is applied to remove redundant candidate boxes, retaining only those with the highest confidence for classification. This optimization enhances the speed of recognition computation. In this study, the grid resolution was set at 9 pixels per grid, and the NMS threshold was fixed at 0.5.

**Figure 2:**
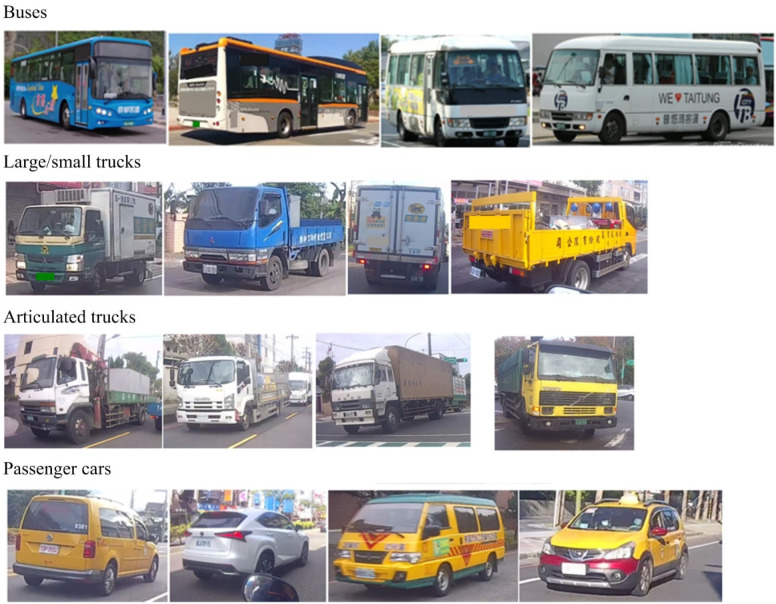
Photographs of various vehicle types were captured from multiple angles (e.g., front, side, and rear) to be used for YOLO model training and validation.

Approximately 2000 photos were collected for this study, as shown in Figure 2, with a roughly equal number of images for each vehicle type. All images were resized to a standardized 256 × 256 pixel size to prevent training disruptions caused by oversized images. These photos were divided into a training set and a validation set. To ensure the machine learning algorithm could recognize objects from various perspectives, vehicle images captured from different angles (e.g., front, side, rear) were included as training samples. Supervised learning was employed, with the target objects in each segmented image being labeled. The model learns the correlations from the labeled image data.

The loss function is one of the important factors affecting the training process of a model because in the training images, most areas usually do not contain the target objects. Similarly, in most grids of an image, there are no target objects present. This results in the confidence of the grid being reduced to 0 and being unable to converge. The performance and accuracy of object detection is generally evaluated using Intersection over Union (IoU), which calculates the intersection of the ground-truth bounding box (the standard answer) and the predicted bounding box (the prediction result) obtained through deep learning. When the predicted result completely intersects with the ground-truth result, IoU is 1; otherwise, if there is no intersection, IoU is 0. The typical criterion for determining recognition rate is usually based on IoU greater than or equal to 0.5.

YOLO v4 incorporates CIoU (Complete-IoU) loss function, introducing two additional parameters in the loss function: *α* and *ν*. Among them, *α* is a positive trade-off parameter, while *ν* is utilized to measure the consistency of proportions between the predicted box and the target box.

In deep learning, mean Average Precision (mAP) is a crucial indicator for evaluating and validating the performance of a model during training. It primarily utilizes a confusion matrix to visualize the effectiveness of algorithms. From the confusion matrix, the accuracy of each labeled position in the image can be calculated, and then averaged to obtain the average precision (AP). Subsequently, mAP is obtained by averaging the AP of each class.

### 2.3. Distance from Camera to Object

**Figure 3:**
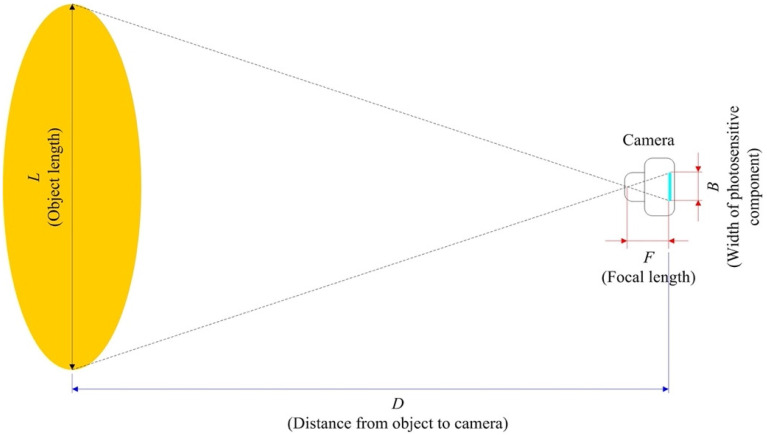
The principle of distance estimation between the camera and the object.

To detect the distance from the camera to the object, the focal length of the camera was first determined using the principle of similar triangles, as shown in Figure 3. Placing an object with a known length L at a distance D in front of the camera, and obtaining the width B of the photosensitive component, the perceived focal length F of the camera can be derived as follows:

(1) $$F\;=\;\frac{D\times B}{\;L}.$$

Once the focal length of the camera lens is obtained, the distance $D_{O}$ between the camera and an object to be detected can be calculated using the following equation:

(2) $$D_{O}\;=\frac{L_{O}\times F}{\;B},$$

where $L_{O}$ is the object length obtained from YOLO v4 object detection.

### 2.4. HWM Model

The development of the HWM model for active safety warning system was carried out using the Python language on Google Colab, https://www.python.org/, leveraging the TensorFlow framework. Its architecture comprises an input layer, seven hidden layers, and an output layer. The input layer receives four inputs, including the vehicle type, the spacing between the motorcycle and vehicle, the speed of the motorcycle, and the distance from the intersection when the vehicle signals to turn. Based on these four different factors, five different levels are assigned, and different scores (5, 10, 15, 20, and 25) are given according to the degree of impact on the rider’s risk for each level, as listed in Table 1. The output is the sum of the scores for the four factors. For instance, if an articulated vehicle is approaching a motorcycle with a speed of 60 km/h, the distance between them is 3 m. When the distance from the intersection is 6 m, the articulated vehicle signals a right turn. In this situation, the danger score reaches 80. The motorcycle rider must decelerate to avoid accidents caused by the blind spot created by the radius difference between inner wheels when the articulated vehicle turns. The higher the score, the greater the danger, and the more cautious the rider should be.

The hidden layers are the pivotal component in HWM, enabling them to learn complex data representations. The HWM in this study consists of seven hidden layers with neuron counts of 18, 36, 72, 36, 18, 9, and 3, respectively. Rectified Linear Unit (ReLU) activation functions were used for all hidden layers.

## 3. Results and Discussion

### 3.1. YOLO v4 Object Detection

The detection targets in this study consist of four categories: buses, large/small trucks, articulated trucks, and passenger cars. Therefore, during training with YOLO v4, the ‘max_batches’ parameter, representing the maximum number of iterations, was set to 8000 (4 vehicle types × 2000 images), and the ‘learning_rate’ parameter was configured to 0.001. Additionally, the computational complexity of YOLO v4 is related to the resolution of the input images. The approximate Giga-Floating Point Operations (GFLOPs) required per inference for the original YOLO v4 model at a 256 × 256 input resolution is around 14.5 GFLOPs.

Different IoU thresholds were employed for comparison. As shown in Table 2, when the IoU threshold is 0.25, the mAP reaches 95.07%, while it is 92.78% when the IoU threshold is 0.5. However, with an IoU threshold of 0.75, the mAP drops to only 57.33%. On the other hand, the precision-recall values are 85% and 82%, respectively, at an IoU threshold of 0.25%, and 88% and 85%, respectively, at an IoU threshold of 0.5, as shown in Table 3. Despite a decrease in mAP of approximately 2.3% when the IoU threshold is set to 0.5 compared to 0.25, precision and recall values are observed to be optimal at the IoU threshold of 0.5.

### 3.2. Influence of Mounting Angle of Camera on Motorcycle

**Figure 4:**
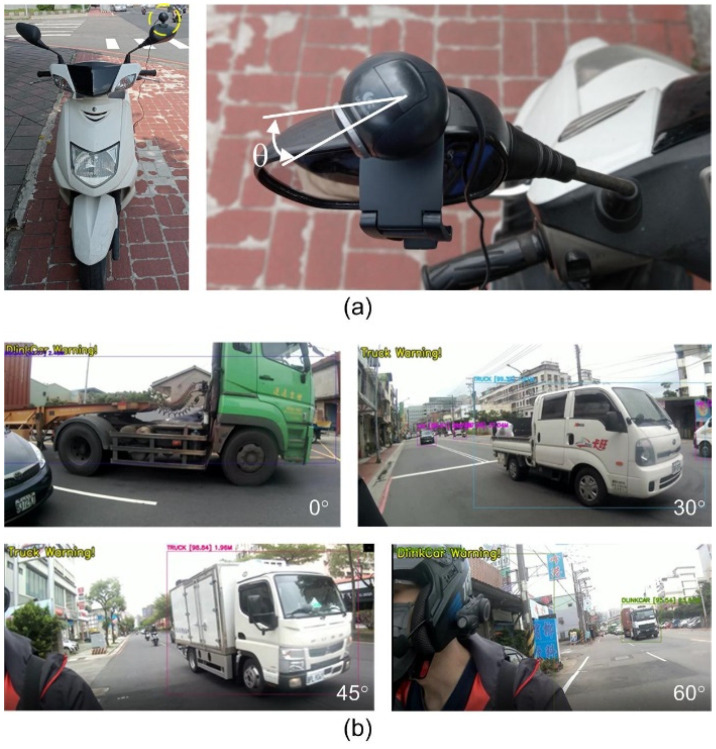
Influence of mounting angle of camera: (**a**) The definition of mounting angle. (**b**) The images captured at angles of 0°, 30°, 45°, and 60°, respectively.

Using the trained YOLO model, actual road videos were captured. This study primarily focuses on right-side driving; therefore, the camera was mounted on the left-side rearview mirror of a motorcycle, as shown in Figure 4a. Because different installation angles of camera result in different timing of capturing images as vehicles enter the camera’s field of view. Thus, the camera was set at different angles relative to the motorcycle’s rearview mirror, namely 0°, 30°, 45°, and 60°, for image observation to obtain the best results after installation.

When the camera is positioned at a 0° degree, it is unable to capture images of vehicles approaching from behind, as shown in Figure 4b. Moreover, when the vehicle is within the camera’s view, this vehicle is already very close to the left side of the motorcycle. Reminding the motorcycle rider at this point may not provide sufficient reaction time if the vehicle suddenly turns, failing to achieve the intended warning effect. When the camera is positioned at a 30° angle, it can clearly capture not only the vehicles traveling in the same direction on dual lanes but also the vehicles approaching from behind on the left side of the motorcycle, allowing this proposed system to analyze and provide advance warning to the motorcycle rider. At a 45° angle, the visible field of view is similar to that at 30°, but buildings adjacent to the lane in the same direction are also captured, constituting invalid detection areas. This can potentially lead to confusion in intuitive interpretation of vehicles in the image. At a 60° angle, compared to 45°, the invalid detection area is even larger, capturing not only the buildings adjacent to the lane and vehicles in parking spaces, but even the rider themselves, which further complicates the visual interpretation of vehicles in the image.

Due to the inclusion of background elements on the right side of the motorcycle at 45° and 60° shooting angles, the YOLO v4 algorithm encountered misclassification errors, specifically by erroneously identifying vehicles parked at the roadside as approaching vehicles. The overall analysis of object detection revealed that at a camera angle of 30°, the highest recognition rate for vehicles was achieved, with an average accuracy of 94.26%. Additionally, grayscale processing of videos was performed to simulate depth sensing, aiming to improve computational speed. However, the algorithmic results showed that the recognition rate for objects was not high, ranging from approximately 65% to 79%.

### 3.3. Distance Estimation

**Figure 5:**
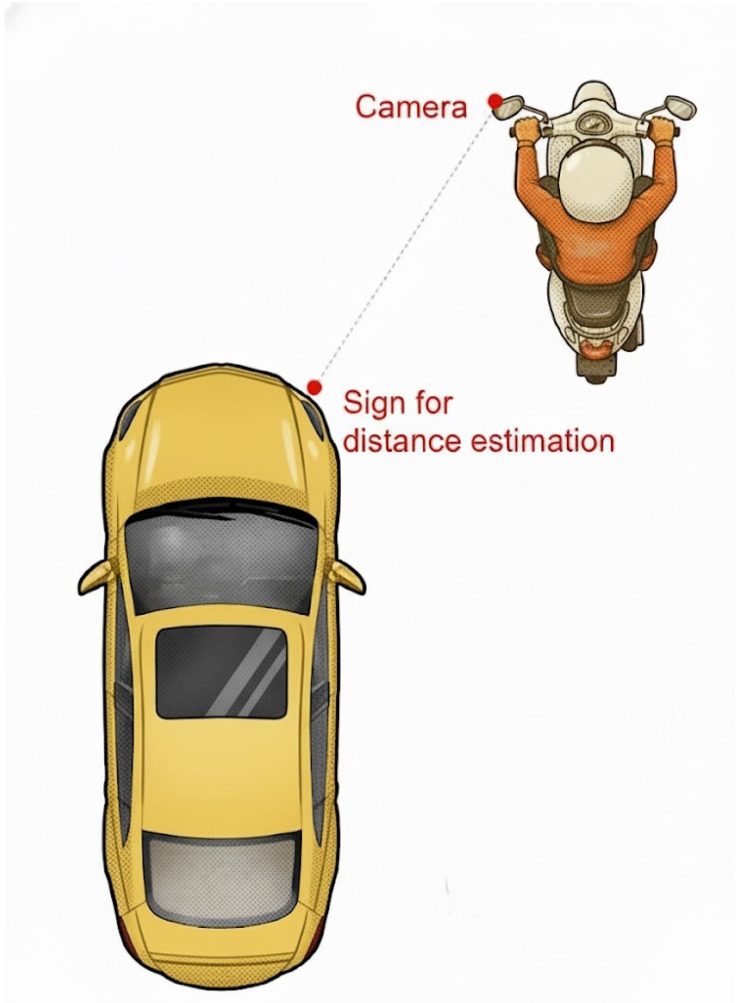
The method for distance estimation with a camera mounted at an angle of 30°.

To validate the consistency between actual distances and those estimated from vehicle images captured by the camera and analyzed using the NVIDIA Jetson TX2, this study employed a camera mounting angle of 30° to detect and compare the distances of four types of vehicles parked either along the roadside or in parking lots. The measurement method is illustrated in Figure 5, with a reference sign placed in front of the vehicle. Both the camera height relative to the ground and the height of the reference sign were maintained at 1.37 m. Distance measurements were conducted for each type of vehicle, with three different vehicles measured per type. Each vehicle was measured in triplicate for the calculation of the average and the root-mean-square deviation (RMSD). The RMSD was subsequently used to determine the error in the estimated distances. Table 4 presents a set of measurement results. Experimental results indicate that the average RMSD errors for all vehicle types are less than 5 cm, demonstrating the accuracy of distance estimation.

### 3.4. Comparison of Object Detection Methods

In recent years, numerous studies have proposed various algorithms based on YOLO to enhance vehicle detection performance. Kang et al. [39] proposed a Type-1 Fuzzy Attention mechanism (T1FA), which utilizes fuzzy entropy to reweight feature maps in order to reduce their uncertainty. This enables the detector to focus more precisely on the object center, thereby effectively improving vehicle detection accuracy. Li et al. [40] proposed a vehicle detection algorithm, YOLO-CCS, which integrates YOLO v5 with a coordinate attention mechanism. This algorithm enhances the network’s ability to focus on vehicles during the feature extraction process. In addition, a faster implementation of the Cross Stage Partial (CSP) Bottleneck, termed C2f, is adopted to reduce information loss and enhance detection performance. Pan et al. [41] proposed a lightweight vehicle detection model based on YOLO, referred to as LVD-YOLO. By integrating the EfficientNetv2 architecture, the model reduces the number of parameters while enhancing feature extraction capabilities. Liu et al. [42] developed the PV-YOLO model based on YOLO v8n for lightweight pedestrian and vehicle detection. In this model, receptive field attention convolution (RFAConv) and a bidirectional feature pyramid network (BiFPN) are employed to simplify the feature fusion process and achieve higher detection accuracy. Zunair et al. [43] compared the detection performance of various object detection algorithms using the RSUD20K dataset, which contains more than 20,000 high-resolution driving-view images, and concluded that YOLO v6 and YOLO v8 achieved the best overall performance. This study utilized the basic functions of YOLO v4 for vehicle recognition. The comparison of these methods is summarized in Table 5.

### 3.5. Risk Index by HWM Model

**Figure 6:**
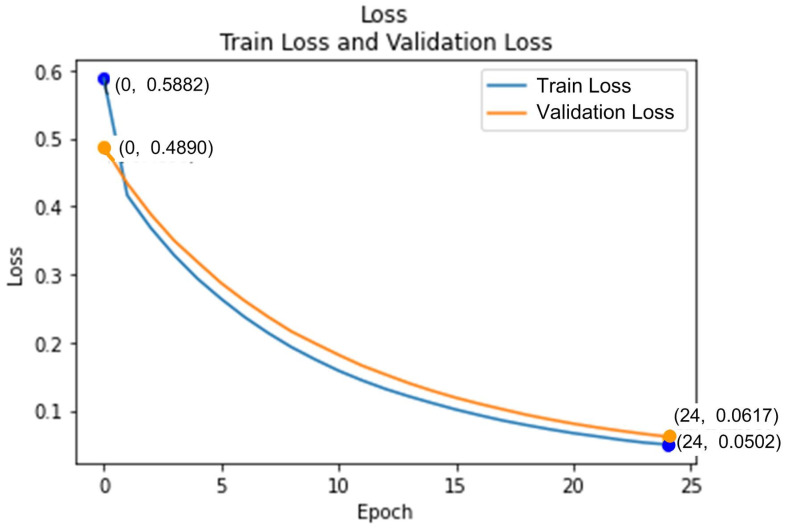
After 24 training epochs, the HWM model achieved a training loss of 0.0502 and a validation loss of 0.0617.

The loss function for various training epochs was estimated using mean-square error (MSE). After 24 training epochs, the training set’s loss function was 0.0502, and the validation set’s loss function was 0.0617, as shown in Figure 6. To evaluate the performance of the HWM model in predicting risk scores, the speed of the motorcycle was maintained at 65 km/h. When an articulated truck approached the motorcycle from behind, the camera measured the distance between the two vehicles to be 2 m, with the intersection approximately 8 m away. The HWM model predicted a risk score of 76.2. Although the motorcyclist could not determine if the articulated truck would make a right turn at the intersection, the predicted risk score was intuitively reasonable. This was because the motorcycle was traveling at a high speed, suggesting that the rider should decelerate to avoid a potential collision due to insufficient braking distance. At present, although a limited number of on-road tests have confirmed the feasibility of the proposed system, more on-road testing is still required to compute performance metrics such as precision, recall, or correlation to further confirm its reliability.

## 4. Conclusions

Numerous data indicate that a significant number of serious accidents involving two-wheeled motorcycles occur when large vehicles make turns. These accidents are often caused by the inner wheel difference and blind spots, resulting in motorcycles beside the larger vehicle being knocked over or even dragged under the vehicle, leading to severe injuries or fatalities. This study utilized the YOLO v4 image recognition technology and HWM models to develop an active safety warning system for two-wheeled motorcycles. The system is capable of recognizing four different types of vehicles and measuring the distance to them. Particularly at intersections, it provides a potential risk score to alert motorcyclists to the need for preventive deceleration, thereby reducing the risk of accidents. Owing to the multilayered architecture of HWM, which confers substantial scalability, these networks can be readily expanded to incorporate additional inputs and subsequently trained on extensive datasets to develop models with enhanced generalization abilities. Therefore, if additional sensors need to be integrated to achieve more advanced safety warning functionalities, this system can be easily adapted to incorporate them. Nevertheless, the current study lacks sufficient exploration into variations in lighting and weather conditions, such as under overcast, at nighttime, or during rainfall. In addition, although the defined scale in the HWM to characterize the level of risk is the simplest and most intuitive approach, other advanced methodologies are worth exploring. These future works are expected to enhance the performance of the active safety warning system for two-wheeled motorcycles.

## Figures and Tables

**Table 1:** Risk score for each level of different factors.

Score	Vehicle Type	Motorcycle Speed (km/h)	Spacing (m)	Distance from Intersection (m)
5	Passenger car	≤40	≥6	≥30
10	Small truck	50	4.5	24
15	Bus	60	3	18
20	Large truck	70	1.5	12
25	Articulated truck	≥80	≤0.5	≤6

**Table 2:** IoU thresholds vs. mAP values.

IoU Threshold	0.25	0.5	0.75
Passenger car	94.91%	92.90%	59.93%
Bus	96.30%	94.57%	57.01%
Articulated truck	93.05%	87.73%	58.70%
Truck	96.03%	95.91%	53.68%
mAP	95.07%	92.78%	57.33%

**Table 3:** IoU thresholds vs. precision-recall values.

IoU Threshold	0.25	0.5	0.75
Ture Positive (TP)	509	527	395
False Positive (FP)	93	75	207
False Negative (FN)	111	93	225
Precision	85%	88%	66%
Recall	82%	85%	85%
Average IoU	68.34%	71.16%	56.16%

**Table 4:** Distance measurement results for all types of vehicles. (Unit: cm).

Vehicle Type	Measured Distance (Average)	Actual Distance	RMSD
Passenger car	236.3	236.0	4.1
Bus	288.3	286.5	4.5
Articulated truck	261.7	260.0	3.5
Truck	211.0	209.5	1.7

**Table 5:** Comparison of object detection methods.

Method	Feature	Reference
YOLO-FA	To reweight feature maps	[39]
(1) YOLO-CCS (2) C2f	(1) Coordinate attention mechanism (2) To reduce information loss	[40]
LVD-YOLO	To reduce the number of parameters	[41]
PV-YOLO	Receptive field attention convolution (RFAConv) and a bidirectional feature pyramid network (BiFPN)	[42]
YOLO v6/YOLO v8	To compare the detection performance using the RSUD20K dataset	[43]
YOLO v4	Basic functions of YOLO	This work

## Data Availability

The original contributions presented in this study are included in the article, and further inquiries may be directed to the corresponding author.
